# Atypical Presentations of Hand, Foot, and Mouth Disease Caused by Coxsackievirus A6 — Minnesota, 2014

**DOI:** 10.15585/mmwr.mm6429a8

**Published:** 2015-07-31

**Authors:** Vicki W. Buttery, Cynthia Kenyon, Stacey Grunewald, M. Steven Oberste, W. Allan Nix

**Affiliations:** 1 Minnesota Department of Health; 2 University of Minnesota at Crookston; 3 Division of Viral Diseases, National Center for Immunization and Respiratory Diseases, CDC

In June, 2014, the Minnesota Department of Health (MDH) was notified of a suspected varicella case in a child aged 2 years. The patient had a generalized rash with relative sparing of the trunk and was hospitalized overnight for treatment of dehydration. The child’s mother, who was near the end of a pregnancy, also had a generalized rash, which included the perineal area. Identifying the cause of the rash was important to determine whether administration of varicella zoster immune globulin was indicated to prevent neonatal varicella (1). *Enterovirus* was detected in specimens from the woman and child by reverse transcriptase-polymerase chain reaction (RT-PCR) testing performed at MDH; partial genome sequencing by CDC showed that both patients were infected with coxsackievirus A6 (CVA6), one of the members of the genus *Enterovirus* that causes hand, foot, and mouth disease (HFMD).

In September 2014, MDH received reports of nine suspected HFMD cases at a college with approximately 1,000 students. Patients ranged in age from 19–47 years and included seven students, one faculty member, and one staff member. Upon arrival at the campus clinic, all had lesions in the mouth, on the palms of the hands, and on the soles of the feet. One patient, aged 20 years, reported having been exposed to a child with HFMD during the previous month; this patient reported the shedding of a thumbnail about 1 month after symptom onset. Throat swabs were obtained from five patients, and an open lesion was swabbed from a sixth. Testing by MDH using RT-PCR identified *Enterovirus* in four of five throat swab specimens and in the swab from the lesion; isolates were subsequently sequenced and identified by CDC as CVA6. There were no complications, and all patients recovered.

HFMD is a common, contagious childhood disease caused by members of the genus *Enterovirus*, usually the coxsackieviruses. HFMD is typically a mild, febrile illness, characterized by mouth sores and a red, sometimes blistery rash involving the palms of the hands and soles of the feet. Nail loss occasionally occurs, often weeks after symptom onset. In the United States, HFMD is commonly caused by coxsackievirus A16. Cases of HFMD with atypical rashes, involving the arms, legs, trunk, perioral regions, buttocks, and genitalia have been recently reported in association with CVA6 (2–4). Although HFMD is most common among children aged ≤5 years, adults can also be infected. However, clusters of HFMD in adults are unusual.

During 2011–2012, an outbreak of HFMD caused by CVA6 occurred in North America. Sixty-three cases were reported to CDC, including 15 among adults. Approximately 50% of the adult patients had reported exposure to children with HFMD (2). The college outbreak reported here might also have begun with an exposure to a symptomatic child. The spread of HFMD among adults in a college setting has not been previously described.

Most cases of HFMD are mild and treatment is supportive, although CVA6 has been associated with more severe disease (2). HFMD is transmitted person-to-person through contact with vesicle fluid, respiratory secretions, and feces. Hand washing and routine disinfection of surfaces help prevent spread. Awareness of unusual features of CVA6, including the occurrence of a varicella-like rash (3,5–7) or a rash with an atypical distribution, can assist health care providers in diagnosing HFMD and recommending appropriate care.
